# Fast tunable metamaterial liquid crystal achromatic waveplate

**DOI:** 10.1515/nanoph-2022-0656

**Published:** 2023-02-16

**Authors:** Majd Abu Aisheh, Mohammad Abutoama, Marwan J. Abuleil, Ibrahim Abdulhalim

**Affiliations:** Ben-Gurion University of the Negev, Beer-Sheva, Israel; Electrooptics and Photonics Engineering, Ben-Gurion University of the Negev, Beer-Sheva, Israel; Electrooptics and Photonics Engineering, ECE-School, Ilse-Kats Nanoscale Science and Technology Center, Ben-Gurion University of the Negev, Beer-Sheva, Israel

**Keywords:** liquid crystals, metamaterials, nanograting, tunable achromatic waveplates

## Abstract

Photonic metamaterials combined with liquid crystals (LCs) for tunability is a great niche for building miniature devices with high performance such as fast flat tunable lenses, tunable filters, and waveplates. Sub-wavelength or nano-grating surfaces are homogenized to uniaxial waveplates with negative birefringence of unique dispersion when the period is less than the wavelength by at least a few times. This uniaxial metasurface, combined with the LC layer, is shown to act as a tunable retardation achromatic waveplate with 8 μm thick LC layer operating over wide spectral and angular ranges, as compared to using two nematic liquid crystal (NLC) retarders of thicknesses on the order of 30–60 μm, when no metasurface is used. Hence the device becomes miniature and 50× faster due to the thinner liquid crystal layer. The silicon nano-grating of 351 nm pitch and 0.282 fill factor is designed and fabricated to operate in the short-wave infrared range (SWIR). Switching between three achromatic retardation levels: full-, half-, and quarter-waveplates is accomplished by changing the applied voltages on the NLC cell with a switching time of a few milliseconds. This device has applications in fast broadband shutters, low coherence phase shift interferometry, ellipso-polarimetry, dynamic control of light intensity, and smart windows.

## 1 Introduction

Metasurfaces are sub-wavelength structured thin surfaces, so for near infrared and visible light wavelengths they are fabricated at the micro- or nano-scales and engineered to interact with light in ways not found in nature [1]. Metasurfaces can be of negative refractive index, in which light passes through bends in the backward direction from the normal axis. One can build the metasurface so that it is inhomogeneous to affect the light wavefront, such as the case with metalenses. Another important function is the optical anisotropy that can be generated by engineering the structure so that the effective refractive index in one direction is different from that in the perpendicular direction. The simplest example is the sub-wavelength grating, which stops diffracting because the period is less than the wavelength [2, 3]. However, it affects the phase of the light passing through it depending on the polarization, so it acts as a uniaxial waveplate. Due to the unique dispersion relations for the form birefringence, it was proposed to build achromatic waveplates using sub-wavelength gratings [4], [5], [6]. One can even build a biaxial plate by careful engineering of the surface. The photonic resonances that can emerge in metamaterials are another topic of high importance for various applications such as filters and fast modulators. The simplest example is the sub-wavelength grating combined with a waveguide layer; here a guided mode resonance appears as a peak in reflection [7]. For thick enough sub-wavelength grating, the resonance can also appear without needing the waveguide layer as the grating starts acting a waveguide [8, 9]. Additional types of resonances can start to appear in sub-wavelength gratings if they are thick enough and with high refractive index contrast such as the bound in the continuum (BIC) ultranarrow resonances [10, 11]. The local optical field at the BIC resonance is enhanced drastically, bringing another dimension to the use of metamaterials in enhanced spectroscopy, low threshold lasing and other applications.

Tunability of metamaterials using electric, magnetic, thermal, or optical stimulus is of high importance to generate fast and compact active devices. Liquid crystals with their strong electrooptic effects can penetrate through micro- and nano-pores and respond to low voltages with very little power consumption are ideal for building fast achromatic and wide field of view metamaterial devices. Several works have demonstrated active LC metamaterial devices such as tunable filtering [9, 12], beam steering [13], light modulation [14, 15], and smart windows [16], [17], [18].

Recently, metasurfaces are under extensive research for developing achromatic tunable metalenses which are flat, compact, and fast tunable that operate at wide continuous spectral ranges and wide field of view [14, 19, 20]. In addition, metasurfaces are used more and more in the field of nanoplasmonics where metallic nanostructures such as nanoholes, nanowires, and nanoparticles are used to enhance optical spectroscopies such as Raman (SERS), fluorescence (SEF) and infrared absorption spectroscopy (SEIRA) [21, 22]. Furthermore, LC metamaterials are used to develop different plasmonic applications, such as plasmonic switches [21] and waveguides [23].

Metamaterials are making a significant breakthrough in developing different optical components by making them smaller, more flexible and with enhanced performance. Among those optical components are the waveplates. Commercial waveplates are made of crystalline materials that pass a long process from precise cut to polish of laser-quality finish up to anti-reflecting coating. Crystalline achromatic waveplates (AWP) are made of multiple crystals of specific thicknesses, which makes them bulky thick retarders and non-practical for applications where compactness is essential [24], [25], [26]. Besides, their achromatic range is not wide enough, usually 200 nm at most and their retardation cannot be tuned. To minimize the form factor, LCs were used in developing different kind of achromatic waveplates. NLCs were used to obtain AQWP in the range of 500–700 nm using two retarders [27], and super AQWP in the range of 400–900 nm using three retarders [28]. Two thin twisted nematic LC retarders were used to obtain zero-order achromatic quarter waveplate (AQWP) in the visible range that is less sensitive to incidence angle than in NLC [29]. Sub-wavelength gratings were proposed in 1997 to act as AQWPs [5]. This concept was employed to get AQWP in the visible [4], [5], [6] and mid-infrared ranges [30]. A significant limitation of the aforementioned waveplate designs is their inability to tune between different phase retardation levels or between different wavelength ranges. This problem was resolved by the design proposed by Abuleil and Abdulhalim [31, 32]. The Abuleil-Abdulhalim LC tunable AWP was composed of two retarders of different NLC materials with their optic axis perpendicular to each other. Tunability was achieved by changing the applied voltage on each retarder to switch between quarter waveplate (QWP), half waveplate (HWP), and full waveplate (FWP) modes. Further, they were able to demonstrate operation at more than one wavelength range using the same device. The problem with this device was the thick LC cells used, ∼ tens of micrometers, which slow the responding time to voltage changes with a switching time of a few tens of milliseconds and more. To solve this problem, herein, we demonstrate a tunable LC metamaterial structure designed and built by combining one thin NLC variable retarder with sub-wavelength silicon (Si) nano-grating. The device operates in the short wave near infrared (SWIR) range and achieves switching speeds of a few milliseconds, which in principle, can be made even faster using higher speed LC modes.

## 2 Design and motivation behind using nanograting-LC metamaterial

### 2.1 Design of the Si nano-grating

Sub-wavelength periodic structures can be modeled using different numerical methods such as the scattering matrix approach [33], the finite-difference time domain method [34], and the eigenwaves or the exact modal method [35] and others [35]. The problem with the numerical methods is that they are inconvenient for fitting to the experimental spectrum. Because of that, analytical approaches based on homogenization are commonly used for design applications [36]. When the wavelength is significantly larger than the pitch of the array (*λ* >> *p*), the zero-order approximation of the effective medium theory (EMT) gives approximate values of the effective refractive indices of the transverse electric (TE) and the transverse magnetic (TM) waves through homogenization. The grating can then be treated as a thin film of uniaxial birefringent material with negative birefringence and the optic axis is along the gratings vector (TM polarization direction) [37], [38], [39]. When the pitch is a few times smaller than the wavelength (*λ* > *p*), the second order approximation (known as Rytov approximation) [40] gives better agreement with the exact result for materials used in the IR applications such as silicon. For such materials, the Rytov approximation is used to find the refractive indices of the TM and TE waves [36, 41, 42]. Following Rytov, the two eigen-indices are determined by the two transcendental equations [36]

(1) $$\begin{array}{ll} & \sqrt{n_{\text{m}}^{2}-n_{\text{TE}}^{2}}\tan\left(\pi \frac{p\left(1-f\right)\sqrt{n_{\text{m}}^{2}-n_{\text{TE}}^{2}}}{\lambda }\right)\\ & \;=-\sqrt{n_{\text{g}}^{2}-n_{\text{TE}}^{2}}\tan\left(\pi \frac{pf\sqrt{n_{\text{g}}^{2}-n_{\text{TE}}^{2}}}{\lambda }\right) \end{array}$$

(2) $$\begin{array}{ll} & \sqrt{n_{\text{m}}^{2}-n_{\text{TM}}^{2}}\tan\left(\pi \frac{p\left(1-f\right)\sqrt{n_{\text{m}}^{2}-n_{\text{TM}}^{2}}}{\lambda }\right)\\ & \;=-\frac{n_{\text{m}}^{2}}{n_{\text{g}}^{2}}\sqrt{n_{\text{g}}^{2}-n_{\text{TE}}^{2}}\tan\left(\pi \frac{pf\sqrt{n_{\text{g}}^{2}-n_{\text{TE}}^{2}}}{\lambda }\right) \end{array}$$

Where *n*_m_ is the refractive index of the material between the grating lines, *n*_g_ is the refractive index of the grating material, *n*_TE_ is the refractive index of the ordinary wave (TE), *n*_TM_ is the refractive index of the extraordinary wave (TM) and *f* is the fill factor of the grating (linewidth divided by the pitch). The tangential terms in Eqs. (1) and (2) can be expanded into power series. Considering the first order of the expansion and plugging it in the transcendental equations gives the zero-order approximation in *p*/*λ* for the refractive indices [36]

(3) $$\begin{array}{ll} & n_{\text{TE0}}=\sqrt{n_{\text{m}}^{2}\left(1-f\right)+fn_{\text{g}}^{2}},\\ & n_{\text{TM0}}=\frac{n_{\text{m}}n_{\text{g}}}{\sqrt{n_{\text{g}}^{2}\left(1-f\right)+fn_{\text{m}}^{2}}}, \end{array}$$

For the third order in the expansion and second order in *p*/*λ* [36]

(4) $$n_{\text{TE}2}={\left\{n_{\text{TE0}}^{2}+\frac{1}{3}{\left[\frac{\pi f\left(1-f\right)p}{\lambda }\right]}^{2}{\left(n_{\text{g}}^{2}-n_{\text{m}}^{2}\right)}^{2}\right\}}^{1/2}$$

(5) $$\begin{array}{ll} n_{\text{TM}2} & =\left\{n_{\text{TM0}}^{2}+\frac{1}{3}{\left[\frac{\pi f\left(1-f\right)p}{\lambda }\right]}^{2}\right.\\ & \;\times {\left.{\left(\frac{1}{n_{\text{g}}^{2}}-\frac{1}{n_{\text{m}}^{2}}\right)}^{2}n_{\text{TM0}}^{6}n_{\text{TE0}}^{2}\right\}}^{1/2} \end{array}$$

**Figure 1:**
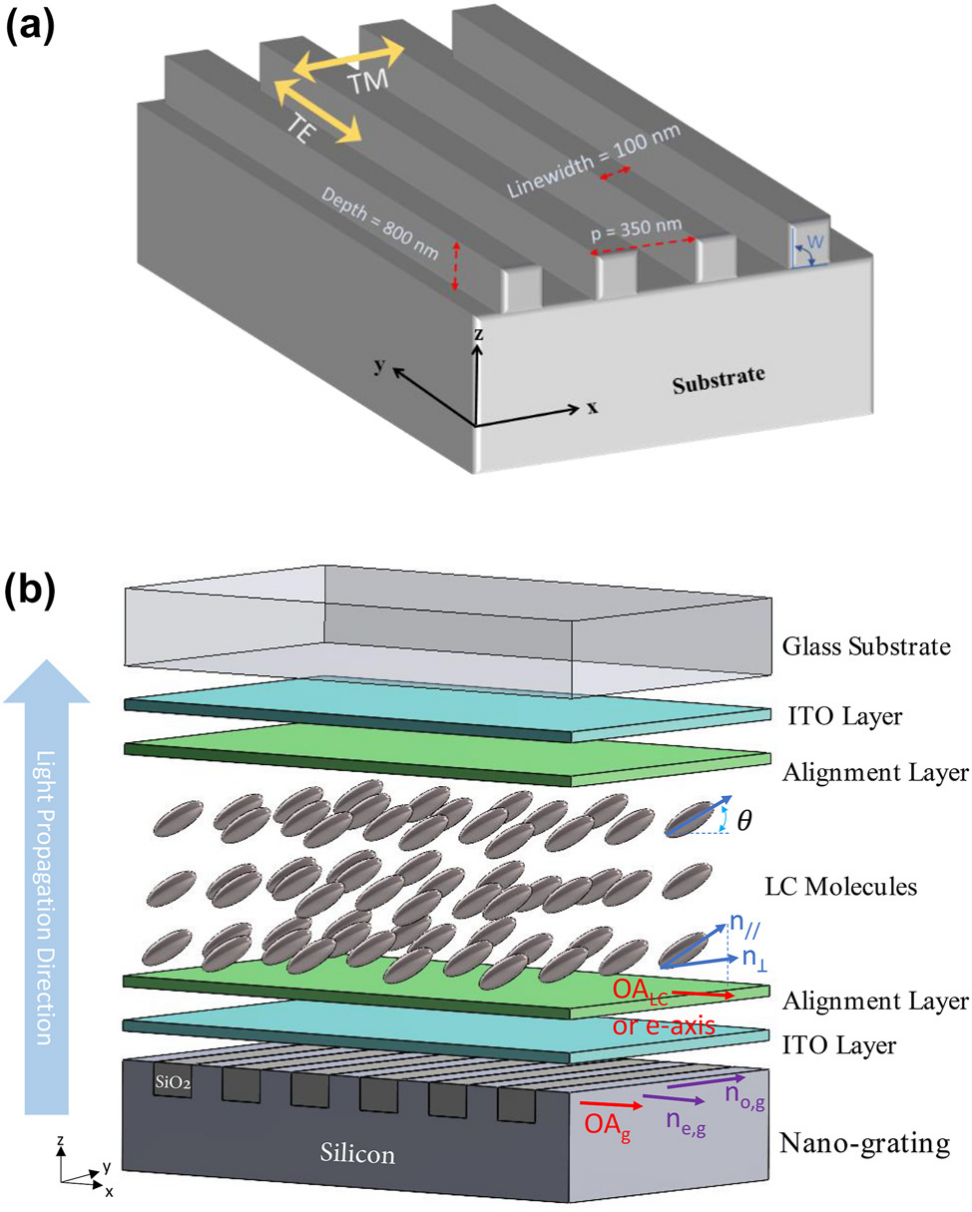
Schematic drawings of the grating structure and the LC metamaterial device. (a) The grating geometry and the direction of the electric field of the TE and TM eigenwaves showing the optimum design parameters, (b) The designed ideal metasurface combined with the LC layer, transparent electrodes and alignment layers.

To decide on whether to use nanograting or other structures and which materials to use, the Rytov 2nd order approximation was used. Although, as it will be seen later, more rigorous calculations are required for comparison to the experimental data. The idea behind the use of nanograting is stimulated from the unique dispersion relation curve of the form birefringence that one can design so that it is almost parallel to the dispersion curve of the LC birefringence at a specific voltage. Figure 1(a) shows that the grating becomes a negative birefringence uniaxial waveplate with the optic axis along the grating vector. It will be shown in Section 3.1 that retardation of nanogratings is sensitive to the wall angle *W* shown in the same figure. The problem of how to build the LC-nano-grating structure caught our attention to consider several design criteria. First, the LC molecules tend to align along the grating lines direction [43] to minimize the free surface energy. This causes the retardation of the LC layer to add to the nanograting retardation; therefore, no dispersion compensation is expected. An ideal device will be the one shown in Figure 1(b) in which the grating is made of two materials such as Si and SiO2, and then covered with indium tin oxide (ITO) as a transparent electrode and an alignment layer. Then the LC optic axis will be oriented parallel to the grating vector, and because the nanograting has negative birefringence, the net retardation will be the difference between the two absolute values of the LC and grating retardations. Hence dispersion compensation can be achieved. Silicon was chosen due to its high refractive index, which helps achieve high retardation with shorter grating depth and transparency in the infrared range. In Figure 1(b), *n*_⊥_ and *n*_∣∣_ are the perpendicular and the parallel refractive indices of the LC molecules, respectively, *n*_o,g_ and *n*_e,g_ are the ordinary (TE) and the extraordinary (TM) refractive indices of the Si grating, respectively, OA_LC_ and OA_g_ are the optical axes of the LC cell and the Si grating, respectively, and *θ* is the tilt angle of an LC molecule. *n*_⊥_ and *n*_o,g_ point in the *y*-direction, and *n*_∣∣_ is oriented in the *xz*-plane. The OA_LC_ is in reality along the molecular axis and it is variable with the distance between the substrates, however it remains in the *xz* plane and what is important is its projection on the *x* axis which defines the extraordinary ray polarization. Therefore in Figure 1(b) we wrote OA_LC_ or *e*-axis which is in fact representing the projection of the LC optic axis on the *x* axis.

Another essential design criterion is the period of the nanograting. The smaller the pitch, the better the Rytov 2nd order approximation, so the better the fit between the simulated results and the experimental ones. However, the birefringence becomes smaller, meaning there is an optimum grating period and duty cycle. On the other hand, manufacturing larger period gratings are more accessible, in particular when a high aspect ratio is required. The minimum grating period that our etching system could achieve was around 350 nm, and to have the best surface finish, the linewidth of the grating is recommended to be 10% of the 800 nm depth of the grating. The calculated retardation using Rytov model was compared with the retardation of the available NLC (Merck BL036).

**Figure 2:**
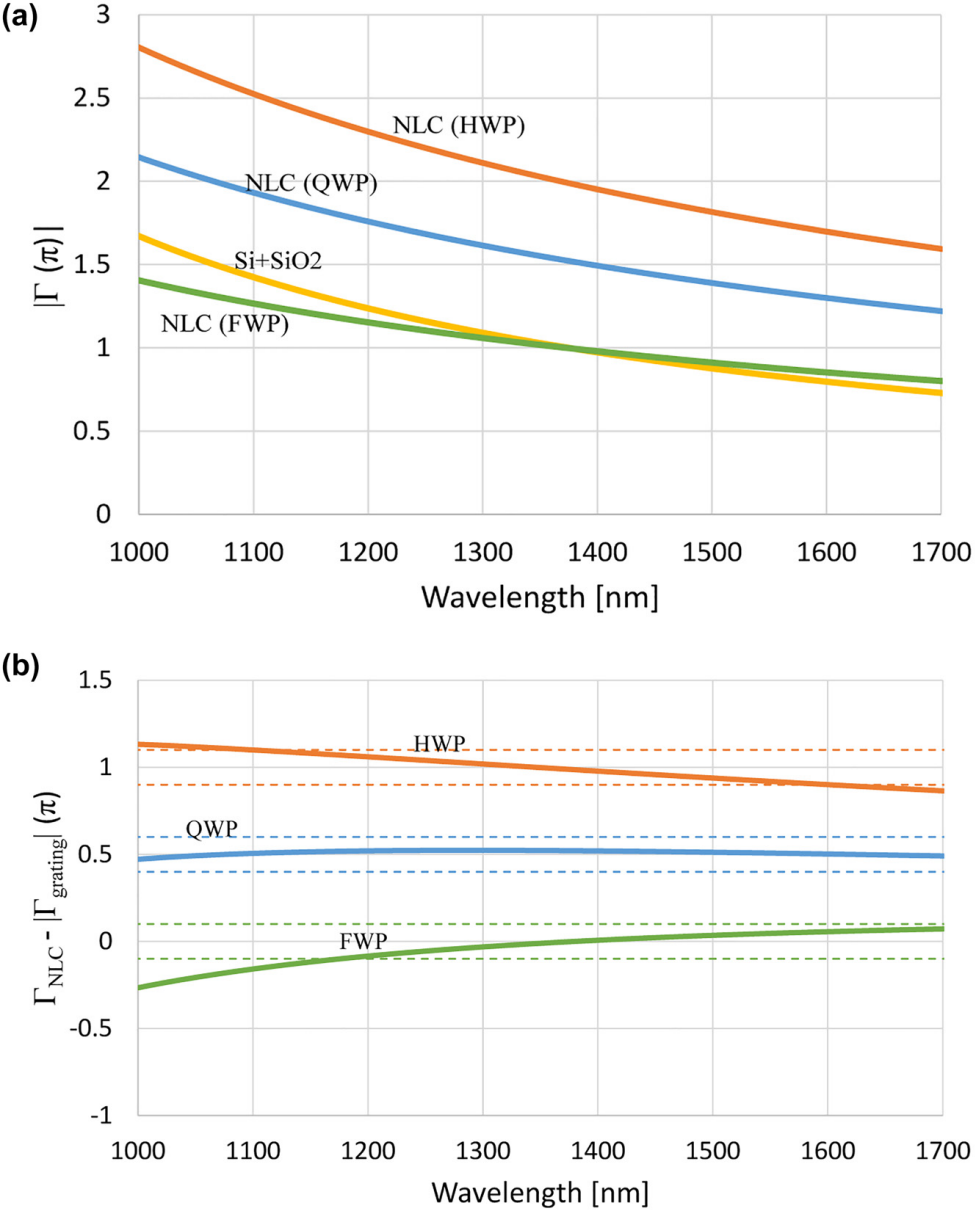
Designed phase retardation dispersion curves. (a) Phase retardation of the designed nanograting and the retardation of an 8 μm LC BL036 at three different voltages which when combined with the nanograting give the three AWP modes, the absolute value was used because the retardation of the grating is negative. (b) Retardation of the combined metasurface at the three AWP modes. The achromatic range is defined between the dashed curves which designate deviation by 0.1π from the desired retardation level.

Figure 2(a) shows the retardation of an 8 μm NLC cell at three different LC molecule tilt angles assuming the device is a uniform waveplate compared with the retardation of the grating. It is noticeable that the FWP has almost an identical retardation curve to that of the grating, and the curves for the QWP and HWP modes are shifted by *π*/2 and *π*, respectively. The retardation calculation of NLCs is explained in detail in Section 3.1. The simulation result shows that tunable AWP can be achieved by combining the nanograting with the thin NLC cell. Figure 2(b) shows the retardation of the designed metasurface when the NLC molecules are aligned so that their optic axis is parallel to the optic axis of the gratings (along the grating vector). This shows that the total phase retardation is zero for the FWP case, *π*/2 for the QWP case, and *π* for the HWP case. It should be noted that the Si-SiO2 grating retardation is not affected much by the fact that the LC layer is directly on top of it because of several reasons: (1) at normal incidence and in the infrared, the Fresnel phase both for the ordinary and extraordinary waves does not change upon transmission, (2) the LC extraordinary refractive index n_e_ at the interface is almost constant under strong anchoring conditions, (3) the Fresnel reflection of the e-mode will be negligible because n_e_ of the LC is close to that of the Si-SiO2 grating. Due to the complicated fabrication process of such a device and to demonstrate the proof of concept, instead of building an ideal Si-SiO2 grating with ITO and alignment layer, the fabricated Si-grating was assembled not in an integrated manner with the LC cell to avoid LC surface alignment effects. So the parameter *n*_m_ in Rytov model is the refractive index of air instead of SiO2. Since the refractive index of air is less than the refractive index of SiO2, the retardation of the Si-air grating is higher than that of Si-SiO2 grating, according to Eqs. (4) and (5). Despite that, the simulation results using Rytov model gave the three AWP modes in the range 1100–2000 nm for such a device, which means that the 8 μm NLC cell is still a valid choice to be combined with the grating. The retardation of the grating was simulated again using COMSOL Multiphysics software, and the results agreed well with the Rytov model.

**Figure 3:**
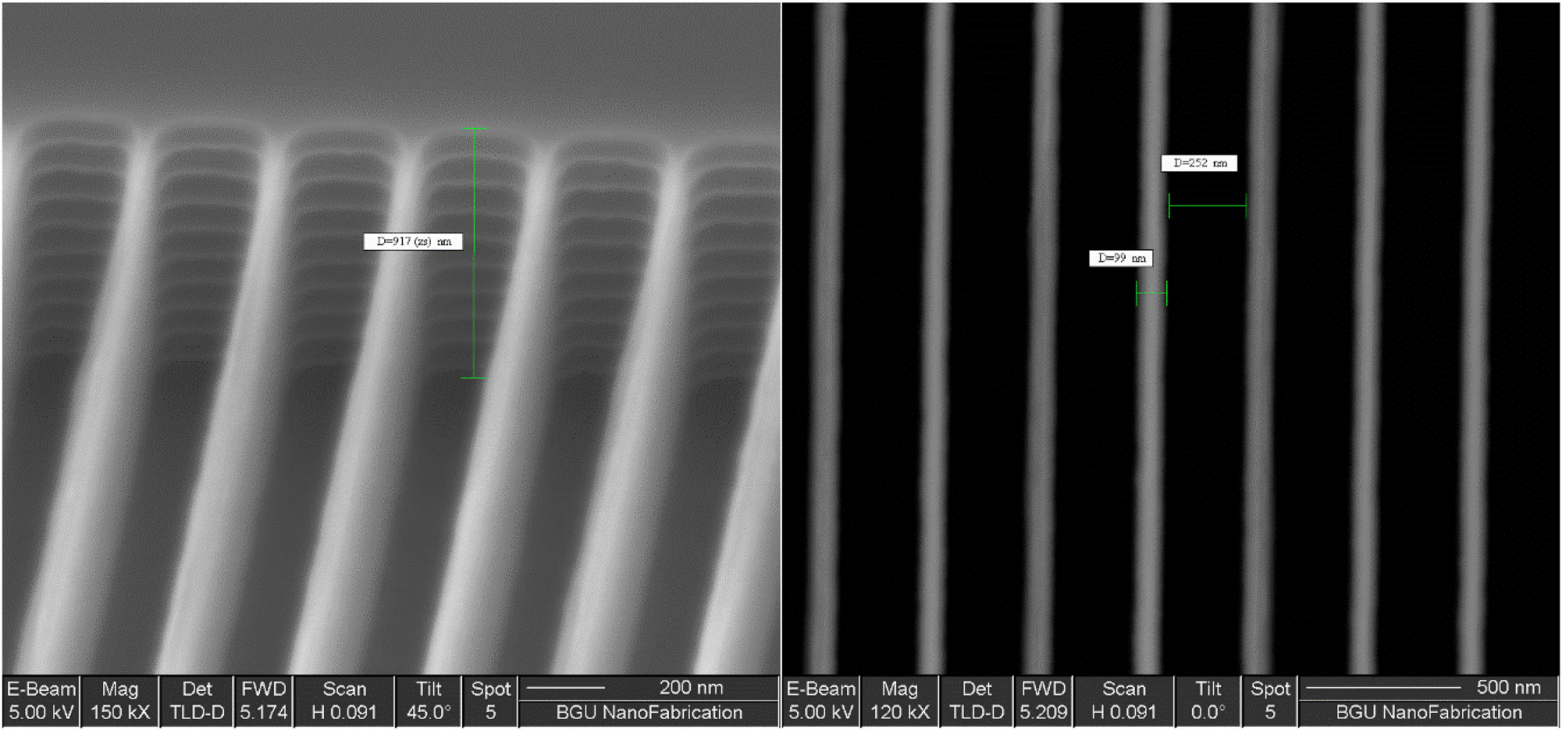
SEM images of the fabricated Si-grating. Pitch is 351 nm which is very close to the designed value (350 nm), depth is 917 nm (designed value was 800 nm), and grating linewidth was 99 nm (designed value was 80 nm), hence the fill factor is 0.282. Note that in (a) the image is titled in purpose to enable measuring the height.

The fabrication process is explained in Section 3.1; however, we present in Figure 3 the SEM images to show the obtained grating parameters. The SEM images cannot be very sharp through the whole thickness of the grating because its depth is larger than the depth of field of the electron microscope. For estimating the period, and top linewidth it is good enough, however the depth was estimated from the side as seen in Figure 3(a).

### 2.2 Choosing the thickness of the NLC cell

After the fabrication of the Si grating substrate, the final thickness of the BL036 NLC cell was determined using the measured retardation data of the grating substrate. The NLC cell was designed to have anti-parallel alignment where the tilt angle (*θ*) profile is uniform throughout the cell at zero voltage. Knowing that retardation is given as Γ = 2*πd*Δ*n*/*λ*, where *d* is the thickness of the cell and Δ*n* is the birefringence of the material, the birefringence will be dependent on the tilt angle of the LC molecules which is a function of the applied voltage

(6) $$\Delta n=n_{\text{e}}-n_{\text{o}}=\frac{n_{⊥}n_{\|}}{\sqrt{n_{\|}^{2}+\left(n_{⊥}^{2}-n_{\|}^{2}\right)\cos^{2}\theta }}-n_{⊥}$$

**Figure 4:**
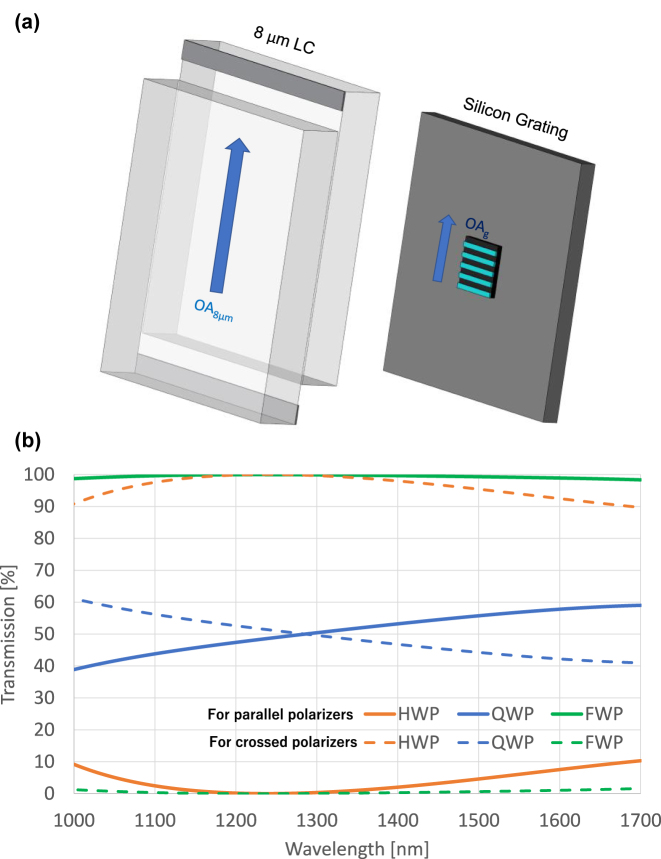
Schematic showing the optic axis orientations of the nanograting and LC layer and the resulting transmission spectrum simulated using measured retardation of the nanograting. (a) Orientation of the system. Optic axies of the NLC cell and the Si grating in the system, (b) simulation results using the measured retardation.

Since the molecules at the boundaries are fixed, a director profile arises when voltage is applied. Thus, the tilt angle varies locally with the coordinate between the glass substrates and the applied voltage. In this case the total birefringence needs to be calculated by integrating over the LC director profile. For the initial design, it is good enough to assume uniformly switching waveplate and simply varying the birefringence by varying the tilt angle as the tilt angle at the center of the LC cell. The true central tilt angle at the particular voltage will be larger than the one used in the uniform waveplate design because of the non-uniform profile. To obtain better dispersion compensation, for example to compensate for fabrication deviations from the grating design, one may use an additional thin LC retarder at fixed voltage with optic axis orientation either parallel or perpendicular to the grating optic axis depending on whether an increase or decrease of the retardation is required. The LC device has its optic axis at zero voltage, or its projection at any voltage parallel to that of the grating (Figure 4(a)).

The performance of the device can be tested in a transmission setup when it is sandwiched between a polarizer oriented at 45° to the optic axis of the grating and analyzer at an arbitrary angle. The system can be represented using the Jones matrix, where the light entering the system through the polarizer is assumed to be linear in the *x*-axis represented as $J_{\text{in}}=\left(\begin{array}{c} 1\\ 0 \end{array}\right)$ , the analyzer matrix is designated by *A*(*φ*) where *φ* represents the orientation of the analyzer axis to the polarizer. The output Jones vector of the system is then given as

(7) $$\begin{array}{ll} J_{\text{out}} & =A\left(\varphi \right)Ret_{\text{LC}}\left(+45{}^{\circ}\right)Ret_{\text{g}}\left(-45{}^{\circ}\right)J_{\text{in}}\\ & =A\left(\varphi \right)\left(\begin{array}{c} \cos\left(\Gamma_{\text{LC}}-\Gamma_{\text{g}}\right)/2\\ -\text{i}\sin\left(\Gamma_{\text{LC}}-\Gamma_{\text{g}}\right)/2 \end{array}\right) \end{array}$$

where Ret_LC_ and Ret_g_ represent the Jones matrices of the LC cell, and the Si grating as uniaxial waveplate, respectively, while Γ_LC_ and Γ_g_ represent their corresponding retardations which are dependent on the voltage and the wavelength. When the polarizers are parallel (*φ* = 0), the transmission is given as $\cos^{2}\left(\Gamma_{\text{LC}}-\Gamma_{\text{g}}\right)$ , and when the polarizers are crossed, the transmitted is $\sin^{2}\left(\Gamma_{\text{LC}}-\Gamma_{\text{g}}\right)$ . QWP is obtained when Γ_LC_ − Γ_g_ = *π*/2 + *mπ*, where *m* is an integer. In this case, the linear polarization will become circular, and the transmission will be 50% between both crossed and parallel polarizers. HWP is obtained when Γ_LC_ − Γ_g_ = (2*m* + 1)*π*, the polarization of the light will rotate by 90°, and transmission will be 100% between crossed polarizers and 0% between parallel polarizers. When Γ_LC_ − Γ_g_ = 2*mπ*, the system is FWP where the polarization of the light at the exit will be similar to that at the entrance so 0% transmission is obtained when the system is between crossed polarizers and 100% transmission if the system is between parallel polarizers. In the design, the Cauchy dispersion relations for the BL036 NLC were taken from [44]. The dispersion of BL036 and the measured retardation data of the Si grating were inserted in MATLAB algorithm that changes thickness of the NLC cell and its central tilt angle until minimum root mean square error (RMSE) for the thinnest cell is obtained for the transmission results to the QWP case, that is 50% transmission through the whole SWIR range (1000–1700 nm). First, the measured retardation data, which was different from the calculated one using Rytov approximation as shown in the previous section, was used to obtain the three phase retardations using a single NLC cell. Results showed the thinnest cell thickness that give AWP in the three modes was 8 μm for the cell optic axis aligned parallel to the Si grating optic axis as shown in Figure 4(a). The simulated transmission results for the three AWP cases are shown in Figure 4(b). The algorithm was run again for cell of the same thickness, but this time using the calculated retardation by Rytov model to compare the experimental results with the theoretical ones following Rytov equations. Results imply that the theoretical Rytov calculation gives better achromatism for the QWP and HWP modes; it will be shown in Section 3.1 that this is due to the wall angle of the grating, which means that the good surface finish of the grating gives better retardation by the grating.

## 3 Device buildup and characterization

### 3.1 Nano-grating fabrication and characterization

The Si substrate was cleaned using the piranha solution (sulfuric acid and hydrogen peroxide), then rinsed with DI water and dried with nitrogen gas followed by O2 plasma ashing for 10 min. After cleaning, the sample was spin coated with commercial PMMA A6 950 K at 4000 rpm and prebaked on a hot plate at 180 °C for 2 min before exposure by electron beam lithography (EBL) system. The exposure was carried out with a Raith EBPG 5150 EBL system with 400 μC cm^−1^ exposure dose at a current of 5 nA. After exposure, the resist was developed for 50 s in MIBK:IPA (1:1) and rinsed in IPA for 20 s. Finally, the sample was post-baked at 100 °C for 1 min on a hotplate, followed by O2 plasma for 12 s. The deep silicon etching was performed using the Bosch process. The Bosch process, consists of 10 cycles of alternating etching and deposition steps, was carried out with PlasmaPro 100 Estrelas DRIE system from Oxford Instruments. The Bosch main recipe parameters for deep Si etching are summarized in the following Table 1.

**Table 1:** Bosch main recipe parameters for deep Si etching.

Step name	Process time [ms]	APC pressure [mTorr]	HF power [W]	ICP power [W]	SF6 [sccm]	C4F8 [sccm]	Table temperature
Deposition	600	20	5	1250	5	120	20
Break main	325	30	60	2000	160	5	20
Etch	200	80	0	2500	360	5	20

**Figure 5:**
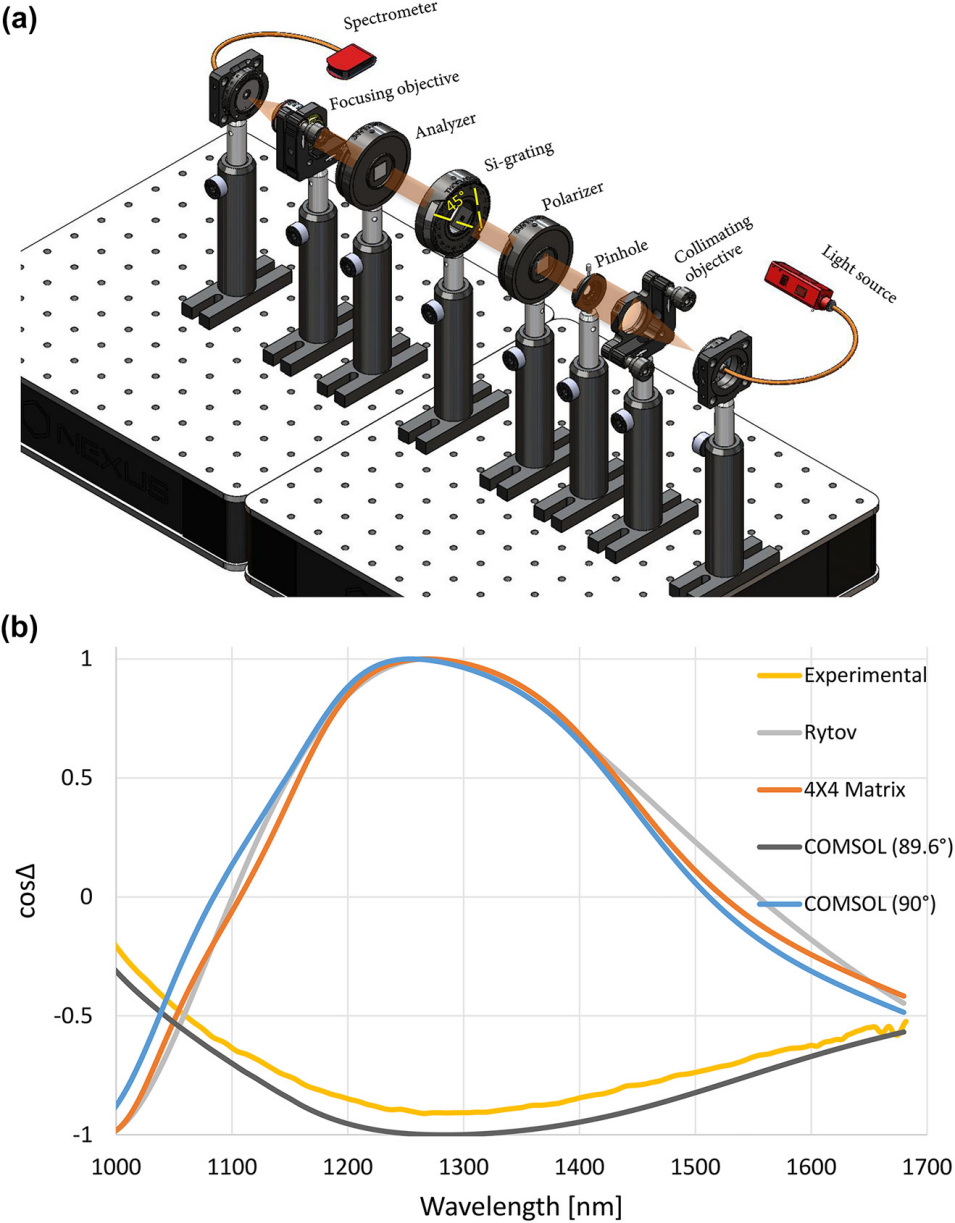
Schematic of the measurement setup, the resulting cosine function of the retardation both experimetal and simulated using different simulation tools. (a) Retardation measurement setup, (b) The cosine function results of the retardation (cos Δ) found experimentally, using Rytov model, 4 × 4 matrix method, and using COMSOL for wall angles of 90° and 89.6°.

The final fabricated Si grating is shown in Figure 3 with all its parameters. Phase retardation was measured by placing the Si-grating with the optic axis (grating vector) at fixed angle of 45° to the polarizer axis as shown in Figure 5(a), then the transmitted light passed through an analyzer parallel to the polarizer before reaching the spectrometer. The ellipse of polarization is described by the ratio between the TM and TE complex amplitudes

(8) $$\begin{array}{ll} \chi & =t_{\text{TM}}/t_{\text{TE}}=\left|t_{\text{TM}}/t_{\text{TE}}\right|\exp\left(\text{i}\left(\varphi_{\text{TM}}-\varphi_{\text{TE}}\right)\right)\\ & =\tan\Psi \exp\left(\text{i}\Delta \right) \end{array}$$

where Ψ = tan^−1^|*t*_TM_/*t*_TE_| is related to the azimuth angle of the polarization ellipse of the transmitted light, and Δ = *φ*_TM_ − *φ*_TE_ is the phase retardation Γ between the TE and TM waves. To calculate the retardation, light intensity is measured at three analyzer angles of −45°, +45°, and 0° relative to the polarizer axis. If light intensity from the source is taken to be 2*I*_0_, the intensity at the output of the setup that reaches the spectrometer is given by the following equation

(9) $$\begin{array}{ll} I & =I_{0}\left[0.5+0.5\tan^{2}\Psi +0.5\left(\tan^{2}\Psi -1\right)\cos2A\right.\\ & \;\left.+\tan\Psi \cos\Delta \sin2A\right] \end{array}$$

The measured light intensities are given by

(10) $$\begin{array}{c} I_{⊥}=I\left(A=-45{}^{\circ}\right)=0.5I_{0}\left[1+\tan^{2}\Psi -2\tan\Psi \cos\Delta \right]\\ I_{45}=I\left(A=0{}^{\circ}\right)=I_{0}\tan^{2}\Psi \\ I_{\|}=I\left(A=+45{}^{\circ}\right)=0.5I_{0}\left[1+\tan^{2}\Psi +2\tan\Psi \cos\Delta \right] \end{array}$$

From these terms, azimuth ratio and phase retardation can be extracted as

(11) $$\tan\Psi =\sqrt{I_{45}/I_{0}}\;\cos\Delta =\left(1/2\tan\Psi \right)\left(I_{\|}-I_{⊥}\right)/I_{0}$$

where *I*_0_ = *I*_‖_ + *I*_⊥_ − *I*_45_ [45, 46].

The measured retardation was compared to the retardation simulated using the Rytov approximation, but significant discrepancy was found (Figure 5(b)). First, we checked using the 4 × 4 matrix approach [47] whether interferences affected due to multiple reflections from the Si surfaces could cause the discrepancy, but this did not resolve the problem. To know the reason for this discrepancy, the problem was simulated using COMSOL software. The wall angle of the grating lines was changed in COMSOL, and it was noticeable that retardation is very sensitive to the wall angle (Figure 5(b)). The best fit with the experimental results was when the wall angle is 89.6° as shown in Figure 1(a). Experimental values of the retardation were used to design the thickness of the NLC cell again. The strong effect of the wall angle on the retardation maybe attributed to two reasons: (1) the effective fill factor is larger since the maximum retardation is obtained at fill factor equals 0.5, (2) multiple reflections between the grating lines start to take place when the wall angle becomes different from 90°, thus larger path length and *π* phase shifts start to appear at each air-Si reflection. The latter is important particularly with deep gratings as in our case, which then needs the rigorous simulations using COMSOL as the Rytov homogenization approach fails under such multiple reflections between the Si walls.

### 3.2 NLC device fabrication and characterization

To produce the NLC cell, glass substrates coated with a conductive ITO layer were cleaned and coated with an alignment layer of SE410 polymer. The alignment layer gives the LC molecules a small pretilt angle (typically ∼6°) after rubbing the surface using a velvet cloth. The pretilt aims to make the device responds faster and achieves the anti-parallel alignment which gives the uniform tilt angle profile through the cell at zero voltage. Microparticles of the desired thickness were inserted between the two glass substrates of the device as spacers on the four corners, and BL036 NLC was filled using the capillary suction method. The cell sides were sealed using UV curing paste and electrodes were connected to apply the voltage that controls the tilt angle profile of the LC molecules, and hence the retardation.

### 3.3 Device assembly characterization with transmission measurement

**Figure 6:**
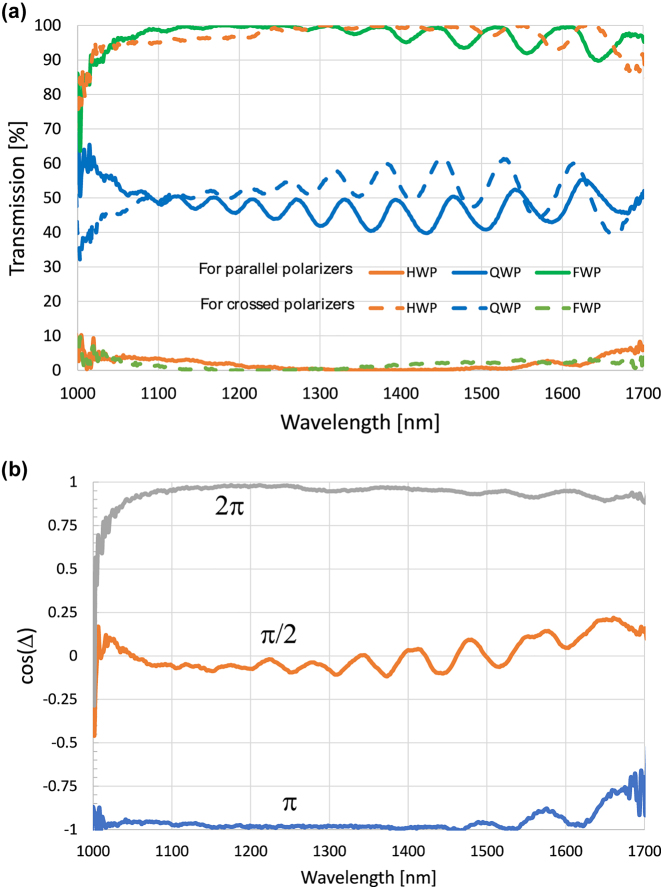
(a) Measured transmission. Measured transmission spectra for the QWP, HWP, and FWP modes between crossed and parallel polarizers, (b) measured cosine of the phase shift. Measured cosine of the phase shift for the QWP, HWP, and FWP modes.

The device assembly of the NLC cell and nano-grating is arranged in the same experimental setup shown in Figure 5(a) between two parallel or crossed polarizers and the transmitted signal is measured with a spectrometer. Light coming from the light source was collimated using a collimation lens, then it passes through a pinhole that minimizes beam size and angular extent, then it passes the polarizer that makes the light linearly polarized along the *x*-axis. The NLC cell and the Si grating were attached to each other and all their optic axes at 45° to the polarizer axis, then the transmitted light passes through the analyzer, and finally, a collecting lens focuses the light at the output to an optical fiber connected to an SWIR Spectrometer (Stellatnet DWARF-Star). Before placing the cell and the Si grating, the spectrometer was calibrated. The dark level was taken when the polarizers are crossed, and the bright reference was taken when the polarizers are parallel, corresponding to 100% transmission. cDAQ-9171 CompactDaq Chassis was used as an automatic square wave function generator that changes the applied voltage automatically, and for every applied voltage, the transmission spectrum was read and recorded. The recorded data was inserted into an MATLAB algorithm that plots transmission profiles at all voltages, records the minimum and maximum transmission readings for each wavelength to use it in normalizing the plots, and measures the RMSE to find the best voltages for the operation as QWP, HWP, and FWP cases. Before normalization, the maximum transmission was around 40%, because of the reflections from the silicon and glass substrates, while the minimum transmission was almost 0% for all the wavelengths. The absolute transmission can be easily increased to over 90% by applying antireflection coatings to the surfaces. However, we did not find it necessary for the proof of concept. The minimum and maximum readings were inserted to the MATLAB algorithm, and it was run again to plot the normalized graphs. To confirm the achromatic waveplate tunable action, we performed both measurements of the retardation as well as transmission between parallel and crossed polarizers. The procedure of measuring the retardation or more accurately cos Δ was explained in Section 3.1. The transmission levels between parallel and crossed polarizers are shown in Figure 6(a) while the cos Δ is shown in Figure 6(b). The QWP, HWP, and FWP modes were achieved at 1.30 V, 1.56 V, and 1.94 V applied on the LC cell, respectively. It is noticed that the plots in Figure 6 start to have high noise at higher wavelengths. This is due to the internal reflections in the glass substrates and the small spaces between the cell and the gratings. Besides, the signal to noise ratio of the spectrometer in this range is smaller.

### 3.4 Response time measurement

**Figure 7:**
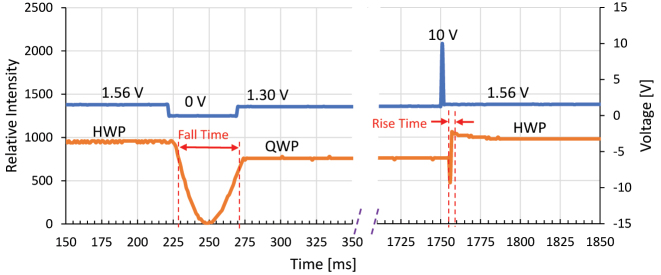
Response time measurement including oscilloscope traces for waveform and signal versus time. The dashed purple lines are to show that the QWP state continued for longer time and therefore broken to better clarify the fall and rise regions.

The concept of using the Si nanograting metasurface combined with a thin NLC retarder instead of using two thick NLC cells is to improve the switching speed of the device, so, the response time is of crucial importance in characterizing this device. To measure the response time, a monochromatic 635 nm laser diode was used instead of the wideband light source in the transmission setup, and the transmitted beam is collected by a photodiode connected to an oscilloscope instead of using a spectrometer. The voltage waveform of 1 kHz square wave was modulated with low frequency pulses that switch the voltage amplitude between the required levels. When the applied voltage is changed between the AWP modes using a function generator, the oscilloscope reading was recorded. The optical rise time is the time it takes for the intensity to change from 10% to 90% of the levels of the intensities corresponding to two states. To improve the response time further, the overshoot drive scheme technique was used [32, 45]. Instead of switching from voltage state 1 (*V*_1_) to voltage state 2 (*V*_2_) immediately, an overshooting voltage in the rise voltage case is applied for a short period of time, and then the system is brought back to *V*_2_. For example, switching from QWP state to FWP state demands to change the voltage from 1.56 V to 1.94 V. The response time, in this case, is around 220 ms. Instead, a higher voltage of 10 V (the maximum the function generator can supply) is applied for 0.92 ms, then the 1.56 V operation voltage is applied. In this case, the rise time becomes 3.2 ms, which is a significant improvement. The same approach is applied when falling from the higher voltage state to the lower voltage state, an undershooting voltage of 0 V is applied for some time, then the state 2 operation voltage is applied. The oscilloscope reading of the latest case with overshoot and undershoot is shown in Figure 7. A summary of response times when switching between the three AWP states is shown in Table 2. One might argue that if using the under/over-shoot method smaller response times can be obtained if the cell thickness and the operating voltages are higher. To test this hypothesis, we prepared three more LC devices of thicknesses 15 μm, 25 μm, and 50 μm all made from BL036 under the same conditions and measured the response times with and without the over/under-shoot method. The results are summarized in the tables in the Supplementary showing that although the operating voltages increases, the response times are still larger than those of the 8 μm cell case.

**Table 2:** Response time when switching between the three AWP modes.

Waveplates	Operationvoltages [V]	Rise time[ms]	Fall time [ms]	Overshootapplicationtime [ms]	Undershooapplicationtime [ms]	Rise time withovershoot [ms]	Fall time withundershoot [ms]
HWP → QWP	1.30 → 1.56	370	700	0.92	48	2.2	45
QWP → FWP	1.56 → 1.94	220	400	0.92	31.2	3.2	28
HWP → FWP	1.30 → 1.94	260	760	1.80	80	3.6	78

## 4 Conclusions

A faster tunable achromatic waveplate based on a combined metamaterial surface and liquid crystal layer operating in the SWIR range is designed and demonstrated experimentally. An 8 μm thin NLC variable retarder with sub-wavelength silicon nanograting metasurface substituted the two thick NLC variable retarders to achieve response times shorter by nearly a factor of ×50. The nanograting was designed according to the Rytov homogenization model and fabricated using an advanced etching method. It was found that retardation of the grating is sensitive to the wall angle, which causes a discrepancy between the homogenization approach and the measurements, believed to be a result of phase shifts associated with multiple reflections between the Si walls as the wall angle deviated from the 90° value. Tunability between AQWP, AHWP, and AFWP was achieved by changing the applied voltage on the NLC cell. The LC devices with the thinnest thickness (8 μm) were found to be the optimum in terms of switching speed. The drive scheme technique was used to improve the switching times to become in the range of a few milliseconds. Faster LC modes are possible to use such as the use of the flexo-electrooptic effect, ferronematic, and ferroelectric LCs. This tunable metamaterial device can be utilized in different applications such as polarimetric imaging, spectroscopic ellipsometry, white light phase shift interferometry, optical coherence tomography, smart windows, and more. We can also design other achromatic metamaterial devices such as tunable achromatic metalenses based on it.

## Supplementary Material

Supplementary Material Details
