# Supplementary material for: Fast tunable metamaterial liquid crystal achromatic waveplate
Source: Nanophotonics. 2023 Feb 16;12(6):1115–27. doi: 10.1515/nanoph-2022-0656 (PMC11501672; doi:10.1515/nanoph-2022-0656)
Supplement: Supplementary file 1 — Supplementary Material Details [file j_nanoph-2022-0656_suppl.docx]

Majd Abu Aisheh, Mohammad Abutoama, Marwan J, Abuleil, and Ibrahim Abdulhalim

Supplementary Information: Fast Tunable Metamaterial Liquid Crystal Achromatic Waveplate

1. Response Time Measurements for Different Cell Sizes

The achromatic waveplate (AWP) was tested using different BL036 LC cell thicknesses to find the optimum thickness that gives the shortest switching speed between the achromatic modes. For thicker cells, the operation voltages are higher, but the difference between the operational voltages increases as well. In addition, the effect of the used overshoot voltage (10 V) is less effective. For the forementioned reasons, the response times between the achromatic modes increase for thicker cells.

Table1: Response time for the 8 $\mu$m cell

| Waveplates | | Operation Voltages [V] | Rise Time [ms] | Fall Time [ms] | | Rise Time with Overshoot [ms] | | Fall Time with Undershoot [ms] | |  |
| --- | --- | --- | --- | --- | --- | --- | --- | --- | --- | --- |
| HWP🡺QWP | 1.30🡺1.56 | | 370 | | 700 | | 2.2 | | 45 | |
| QWP🡺FWP | 1.56🡺1.94 | | 220 | | 400 | | 3.2 | | 28 | |
| HWP🡺FWP | 1.30🡺1.94 | | 260 | | 760 | | 3.6 | | 78 | |

Table 2: Response time for the 15 $\mu$m cell

| Waveplates | | Operation Voltages [V] | Rise Time [ms] | Fall Time [ms] | | Rise Time with Overshoot [ms] | | Fall Time with Undershoot [ms] | |  |
| --- | --- | --- | --- | --- | --- | --- | --- | --- | --- | --- |
| HWP🡺QWP | 2.14🡺2.55 | | 480 | | 740 | | 56 | | 130 | |
| QWP🡺FWP | 2.55🡺3.40 | | 200 | | 490 | | 24 | | 96 | |
| HWP🡺FWP | 2.14🡺3.40 | | 264 | | 830 | | 48 | | 112 | |

Table 3: Response time for the 25 $\mu$m cell

| Waveplates | | Operation Voltages [V] | Rise Time [ms] | Fall Time [ms] | | Rise Time with Overshoot [ms] | | Fall Time with Undershoot [ms] | |  |
| --- | --- | --- | --- | --- | --- | --- | --- | --- | --- | --- |
| HWP🡺QWP | 2.57 🡺3.15 | | 472 | | 1070 | | 108 | | 250 | |
| QWP🡺FWP | 3.15🡺4.25 | | 420 | | 1154 | | 80 | | 188 | |
| HWP🡺FWP | 2.57🡺4.25 | | 430 | | 1520 | | 68 | | 348 | |

Table 4: Response time for the 50 $\mu$m cell

| Waveplates | | Operation Voltages [V] | Rise Time [ms] | Fall Time [ms] | | Rise Time with Overshoot [ms] | | Fall Time with Undershoot [ms] | |  |
| --- | --- | --- | --- | --- | --- | --- | --- | --- | --- | --- |
| HWP🡺QWP | 3.6🡺4.68 | | 600 | | 1260 | | 480 | | 720 | |
| QWP🡺FWP | 4.68🡺6.68 | | 300 | | 640 | | 76 | | 220 | |
| HWP🡺FWP | 3.6🡺6.68 | | 300 | | 1400 | | 88 | | 404 | |

1. Dispersion Relation of the Used Liquid Crystal

Assuming homogeneous molecular orientation through the cell, the dispersion of BL036 is given using Cauchy fit:

$$n_{//,ꓕ}\left( \lambda\right)=n_{//,ꓕ}^{0}+A_{//,ꓕ}\lambda^{-2}+B_{//,ꓕ}\lambda^{-4}$$

The parameters can be found in the following table [1]:

| $n_{ꓕ}^{0}$ | $A_{ꓕ}\left( \mu m^{2} \right)$ | $B_{ꓕ}\left( {10}^{4}\mu m^{2} \right)$ | $n_{//}^{0}$ | $A_{//}\left( \mu m^{2} \right)$ | $B_{//}\left( {10}^{4}\mu m^{2} \right)$ |
| --- | --- | --- | --- | --- | --- |
| 1.513 | 0.0042 | 3.67 | 1.733 | 0.0137 | 25.04 |

The local birefringence is given as:

| $\Delta n=n_{e}-n_{o}=\frac{n_{\perp}n_{\parallel}}{\sqrt{n_{\parallel}^{2}+\left( n_{\perp}^{2}-n_{\parallel}^{2} \right)\cos^{2} \theta}}-n_{\perp}$ |  |
| --- | --- |

where $\theta$ is the effective tilt angle, in the design taken as constant through the cell.

References

[1] Wahle, M. and Kitzerow, H.-S. (2014) “Liquid Crystal Assisted Optical Fibres,” Optics Express, 22(1), p. 262. <https://doi.org/10.1364/oe.22.000262>.
